# Do tight nosebands have an effect on the upper airways of horses?

**DOI:** 10.1002/vms3.1478

**Published:** 2024-06-17

**Authors:** Dominik Scholler, Jana Wittenberg, Yury Zablotski, Anna May

**Affiliations:** ^1^ Equine Clinic of Ludwig Maximilians University Oberschleissheim Germany; ^2^ Equine Clinic of Free University Berlin Berlin Germany; ^3^ Clinic for Ruminants Ludwig Maximilians University Munich Oberschleissheim Germany

**Keywords:** animal welfare, equestrian sports, horse, overground endoscopy, noseband, stress

## Abstract

**Background/Objectives:**

The public perception relating to the welfare of horses involved with equestrian sports is associated with training methods used and the presentation of horses at events. In this context, very tight nosebands, which are intended to prevent the horse from opening its mouth, also attract a lot of attention. Various studies have evaluated the impact of tight nosebands on stress parameters, whereas the effect of tight nosebands on upper airway function is unknown. Therefore, the aim of the study was to use overground endoscopy to evaluate changes in pharyngeal and laryngeal function when a tight noseband is fitted. Moreover, the ridden horse pain ethogram (RHpE) was applied to investigate signs of discomfort (Dyson et al., 2018).

**Study design:**

A randomized, blinded, and prospective study was performed.

**Methods:**

Sixteen warmblood horses consisting of twelve mares and four geldings with a mean age of 11.63 ± 3.53 years were ridden on 2 consecutive days with either loose or tight nosebands (two fingers or no space between bridge of the nose and noseband, respectively) and inserted endoscope in a random order. Videos were taken in a riding arena during a standardized exercise protocol involving beginner level tasks for 30 min in all gaits. For video analysis, freeze frames were prepared and analyzed at the beginning of the expiration phase. Pharyngeal diameter was measured using the pharynx‐epiglottis ratio. Other findings (swallowing, pharyngeal collapse, soft palate movements, and secretion) were also evaluated. Moreover, the RHpE was applied. Descriptive statistics and generalized linear mixed effects models were used. Results with a *p*‐value < 0.05 were considered statistically significant.

**Results:**

While the pharynx‐epiglottis ratio did not change significantly in horses ridden with loose versus tight nosebands, there was an increase in mean grade and total counts of parameters assessed in the pharyngeal region, for example, grade of secretion (1.5 [±SD 0.89] vs. 3.13 [±SD 0.96]; *p* = 0.0001), axial deviation of the aryepiglottic folds (0.29 [±SD 0.73] vs. 1.33 [±SD 1.44]; *p* = 0.01), and pharyngeal collapse (0.69 [±SD 0.87] vs. 1.88 [±SD 1.54]; *p* = 0.005) in horses ridden with tight nosebands. There was no RHpE score above 8 indicating musculoskeletal pain, but the RHpE scores were significantly higher in horses ridden with tight nosebands (*p* < 0.001).

**Main limitations:**

Video quality was limited when horses showed large amounts of secretion. Another limitation was the small number of horses.

**Conclusions:**

Results add to the evidence obtained in other studies that tight nosebands do not only cause adverse reactions based on the RHpE score such as head behind the vertical or intense staring but also contribute to changes in the pharyngeal region, such as increased secretion and collapse of pharyngeal structures. This may provide further support for future decisions regarding regulations on nosebands.

## Graphical abstract

Horses ridden with tight nosebands showed an increase in mean grade and total counts of parameters assessed in the pharyngeal region, for example, grade of secretion, axial deviation of the aryepiglottic folds, and pharyngeal collapse. Horses with tight nosebands showed more adverse reactions when assessed with the ridden horse pain ethogram (RHpE). Results add to the evidence that tight nosebands may impact equine welfare.

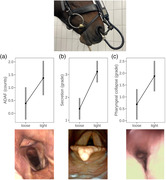

## 1 INTRODUCTION

A central point of public criticism in equestrian sports has been the correct fastening of nosebands, and despite various studies stating the welfare impact, no clear rule has been implemented on an international level (FEI Inside, 2023). Tight nosebands have been reported to cause stress and discomfort in horses, which can be expressed in different physiological and behavioural reactions (Fenner et al., 2016, p. 3). Increases in various stress parameters have been proven, for example, changes in heart rate and heart rate variability and changes in eye temperature and biochemical stress parameters such as cortisol (Doherty, Casey, et al., 2017; Duke, 2017; Mcgreevy et al., 2012; Pérez‐Manrique et al., 2020; Uldahl & Clayton, 2019; Weller et al., 2020). In addition to that, tight nosebands can cause physiological stress responses and inhibit the expression of oral behaviours (Fenner et al., 2016). Reactions often include increased yawning, swallowing, and licking after loosening the tight noseband as a sign of post‐inhibitory reinforcement (Fenner et al., 2016). In summary, many studies concluded that tight nosebands lead to behavioural changes and increased stress parameters in horses.

In the recent past, studies have repeatedly shown that many nosebands are still buckled too tightly, despite the regulations for equestrian events which recommend that there has to be space for two fingers of an adult between the bridge of the nose and the noseband (Mcgreevy et al., 2012). A study by Doherty, Casey, et al. (2017) found that only 7% out of 750 horses examined at competitions had a correctly fastened noseband (Fenner et al., 2016), while in 44% of horses, no finger fitted between the bridge of the nose and the noseband. A more recent study of Canadian riders found that 71% of horses had their nosebands buckled correctly according to the two‐finger rule (Duke, 2017). Furthermore, an evaluation of an online survey on noseband use revealed that there is widespread awareness of the problem regarding tight nosebands and a consensus with respect to the two‐finger rule (Uldahl & Clayton, 2019).

The studies indicate that there is still a problem in the equestrian industry regarding the use of nosebands with either the governing bodies not effectively enforcing the two‐finger rule or the riders knowingly ignoring it.

To evaluate welfare in ridden horses, ethograms are increasingly used to objectively assess parameters that are associated with pain or discomfort. While the ridden horse pain ethogram (RHpE) by Dyson et al. (2018) has been developed to rule out pain or lameness in ridden horses, it has been found that many other aspects of riding can contribute to suboptimal welfare in ridden horses and result in the same behaviours listed in the RHpE (Dyson et al., 2018, Ladewig et al., 2022). According to reviews, the RHpE is impaired by some flaws that limit its use in diagnosing pain as behaviours can be a result of many aspects such as equipment, rider skills, lateralization, and conflict behaviours (Ladewig et al., 2022). The behaviours highlighted in the RHpE do therefore reflect stress in the ridden horse rather than exclusively pain, and it was therefore used in this study to detect discomfort in ridden horses.

The method of overground or exercise endoscopy has been developed to examine the upper airways in ridden horses with respiratory sounds (Franklin & Allen, 2017, Weller et al., 2020). Additionally, the influence of rider interventions on the stability and function of the pharynx and larynx can be visualized (Mcgreevy et al., 2012).

While there are many studies on the outside effects of tight nosebands, the upper airways have not been looked at in detail. Therefore, the aim of the study was to use overground endoscopy to assess changes in pharyngeal and laryngeal function in horses ridden with tight versus loose nosebands. To evaluate adverse reactions and the stress response in the different noseband settings, the RHpE was used. By offering additional evidence for the effect of tight nosebands on horses’ upper airways, this study could help to address the issue in the equestrian world. The hypothesis of the study was that horses ridden with tight nosebands showed discomfort according to the RHpE and changes in upper airway function.

## 2 MATERIALS AND METHODS

### 2.1 Animals

In this study, a group of 16 warmblood horses, consisting of 12 mares and four geldings with a mean age of 11.63 ± 3.53 years, was used. The horses were part of the teaching herd of Bavaria's Main State Stud farm “Haupt‐ und Landgestuet Schwaiganger” in Ohlstadt, Germany. Every horse was subjected to a detailed clinical examination (general examination, clinical lameness examination including flexion tests, cardiac, and lung examination), and only healthy animals were used for this study. Horses were excluded if showing upper airway disorders on endoscopy.

The study protocol was approved by the animal ethics committee of the government of Bavaria (Oberbayern), Munich, Germany (approval AZ ROB‐55.2‐2532.Vet 02‐21‐100, February 2022).

### 2.2 Equipment

#### 2.2.1 Noseband (type, width, and position)

**FIGURE 1:**
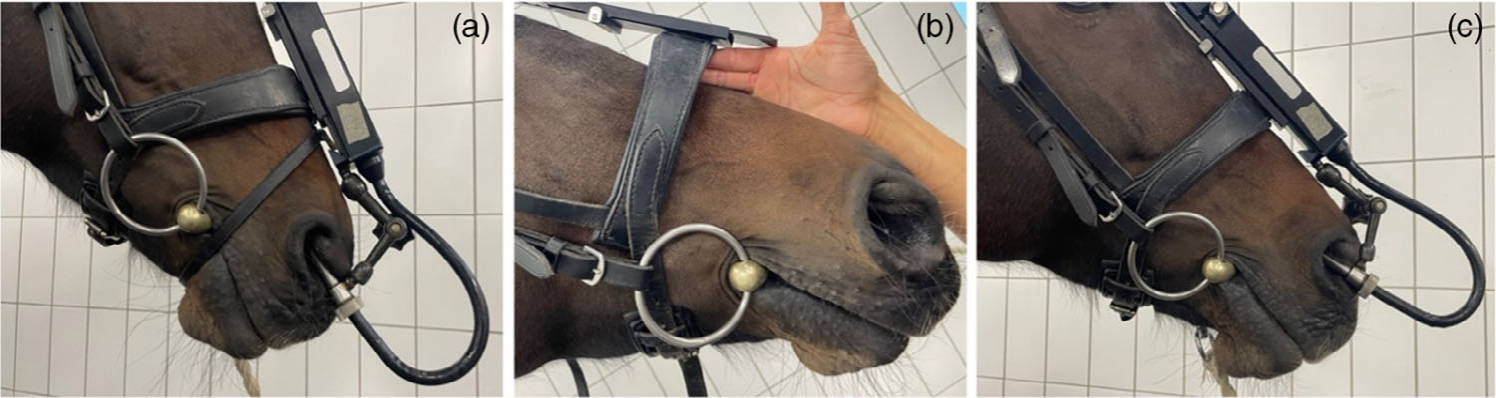
Noseband fastening in the horses prepared for the study: (a) Noseband tightened so that no fingers can be placed between the bridge of the nose and noseband and additional flash strap in place. (b) Two‐finger rule applied for the horses ridden with loose noseband. (c) Loose noseband in place. To not cause a bias during ridden horse pain ethogram (RHpE) video evaluation for the blinded examiners, the horses with loose nosebands were wearing a very loose flash strap.

A flash type noseband was used and fastened similarly in all horses. All horses’ heads had approximately the same size, so they all wore the same bridle and noseband. The noseband was fitted two fingers below the horses’ cheekbone. When ridden with tight nosebands, the nosebands were tightened so much that there was no space between the bridge of the nose and the noseband. The loose noseband was buckled according to the national regulations (two fingers of an adult placed between the bridge of the nose and the noseband, Figure 1).

#### 2.2.2 Overground endoscopy

An overground endoscope by Videomed was used (Videomed Active Airway Endoscope by Videomed GmbH).

The overground endoscope was inserted into the right nostril before riding, and horses became accustomed during the 10‐min warm‐up period. Video sequences of 16 horses were obtained endoscopically, and videos with tight and loose nosebands were taken.

### 2.3 Study protocol

The experiment took place on 2 consecutive days in an indoor riding arena. All horses were ridden twice: one day with endoscope and tight noseband and another day with endoscope and loose noseband. The order in which horses had tight or loose nosebands was randomized. Two amateur riders were riding the horses, and each of the riders rode the same horses twice.

The horses were prepared for this examination for their daily training. Every horse was wearing its normal, accustomed bit (snaffle bits in different sizes) and saddle.

Every horse was ridden according to a standardized protocol involving beginner level tasks for 30 min at all gaits (10 min warm‐up in walk and trot, 10 min standardized riding protocol, and 10 min cool‐down; see supplemental material). The horses’ heads were not placed in a specific position, and riders aimed to ride the horses slightly in front of the vertical. While riding according to the standardized protocol, videos of overground endoscopy and videos for RHpE analysis were taken.

### 2.4 Video analysis

#### 2.4.1 Videos taken by overground endoscopy

For the evaluation of endoscopy in ridden horses, freeze frames from the exercise endoscopy were taken and evaluated using a video‐editing program (EDIUS 8) as described in other studies (Go et al., 2014). Freeze frames were taken at the beginning of expiration phase, and the deglutition phase was excluded.

**FIGURE 2:**
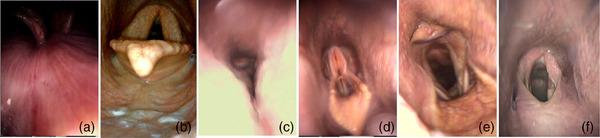
Endoscopic parameters evaluated in this study: (a) swallowing (in counts), (b) secretion (in grades—amount and type, grade 2/4 pictured), (c) pharyngeal collapse (in grades, grade 4/4 pictured) (Boyle et al., 2006), (d) full adduction of the arytenoid cartilages (in counts) (Lane et al., 2006), (e) axial deviation of the aryepiglottic folds (ADAF; in counts) (Lane et al., 2006), and (f) dorsal displacement of the soft palate (DDSP; in counts) (Lane et al., 2006). Definitions of pharyngeal abnormalities based on Boyle et al. (2006) and Lane et al. (2006).

Ten minutes of exercise endoscopy during the standardized riding protocol (trot and canter) was evaluated by two examiners (internal medicine specialists), who were blinded to the noseband type applied and occurrence of swallowing, secretion, pharyngeal collapse, adduction of the arytenoid cartilages, axial deviation of the aryepiglottic folds (ADAF), dorsal displacements of the soft palate (DDSP), as well as soft palate instability were counted (Figure 2).

The definition of the mentioned parameters is shown in Table 1. Grade of secretion was determined using the grading system which was developed by the authors of this study (Table 1), and the grade of pharyngeal collapse was evaluated using the grading system developed by Boyle et al. (2006).

**TABLE 1:** Definitions for the parameters used to evaluate the horses’ pharyngeal function with tight versus loose nosebands during ridden exercise.

Parameter	Definition
Swallowing	Full action of deglutition
Secretion	Amount and type of secretion: grade 0‐4 0: No secretion visible 1: Small amounts of serous secretion in front of larynx, little coming up from trachea, visible during expiration 2: Moderate amounts of serous secretion in front of larynx, secretion coming up from trachea, visible during expiration 3: Large amounts of serous secretion in front of larynx, secretion coming up from trachea, visible during expiration 4: Accumulations of viscous mucus in front of the larynx, permanently visible mucus in trachea (in‐/expiration)
Pharyngeal Collapse (Boyle et al. 2006)	Repeated narrowing of the pharyngeal diameter from dorsal/ventral and left/right, grade 0‐4 (by Boyle et al. (2006)) 0: No pharyngeal collapse 1: Mild, roof of pharynx affected 2: Severe, both walls touching each other 3: Severe, roof and bilateral sides affected 4: Severe, all four walls affected
Full adduction of the arytenoid cartilages (Lane et al., 2006)	Right and left arytenoids are in full contact
Axial deviation of the aryepiglottic folds (ADAF) (Lane et al., 2006)	Prolapse of the fold between the arytenoid cartilage and the lateral margin of the epiglottis on each side – during prolapse partial covering of the arytenoids is visible
Soft palate instability (Lane et al., 2006)	Counting the complete up and down movement (billowing movements) of the soft palate during ridden exercise
Dorsal displacement of the soft palate (DDSP) (Lane et al., 2006)	Caudal free margin of the soft palate displaces dorsal to the epiglottis.

#### 2.4.2 Videos of ridden horses and application of RHpE

During the standardized riding protocol, videos were taken of the horses, and the RHpE was applied as described by Dyson et al. (2018). All videos were independently evaluated by two examiners (internal medicine specialists), who were blinded to the noseband tightness the horses were ridden with. The RHpE score ranges from 0 to 24, while a score of ≥8 is likely to indicate musculoskeletal pain (Dyson et al., 2018).

### 2.5 Measurements of the larynx and pharynx/PE ratio

Measurements were obtained using the graphics software program (ImageJ, rsweb.nih.gov/ij/) as described in other studies (Barton et al., 2023, Go et al., 2014).

**FIGURE 3:**
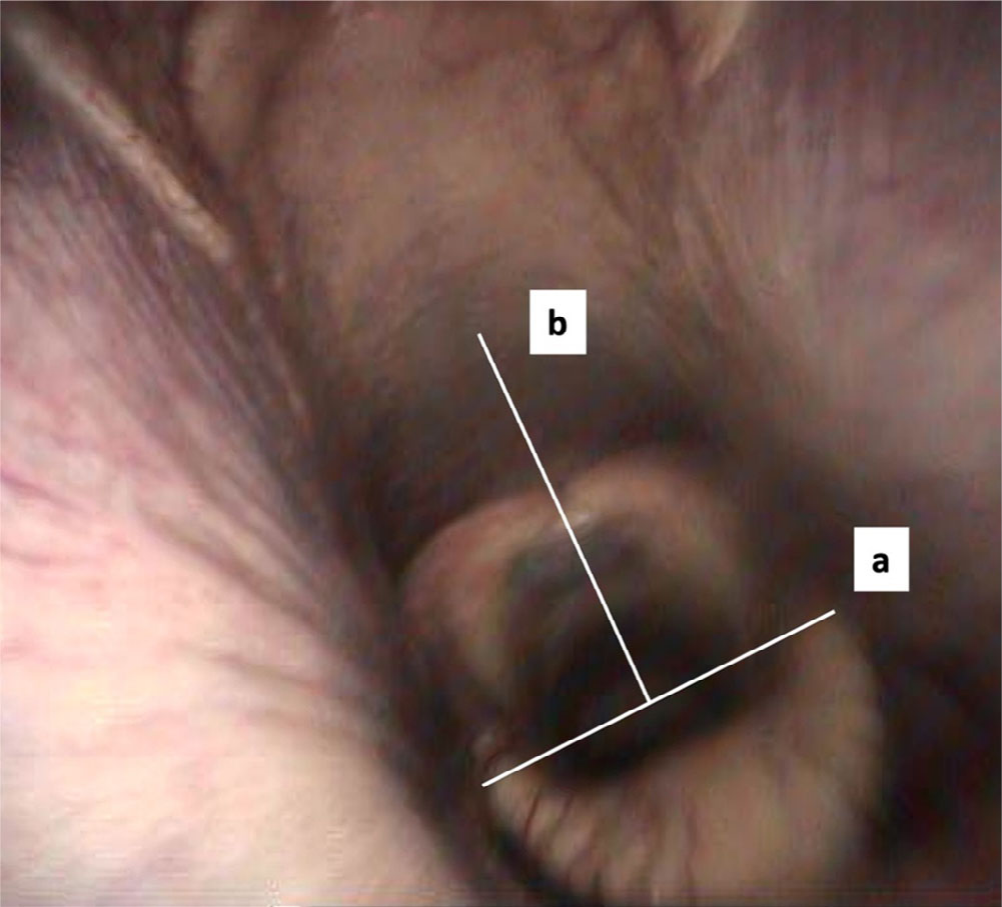
Pharynx‐epiglottis (PE) ratio. Structures on the dorsal pharynx (such as follicles or vascular patterns) and on the epiglottis were used as fixed points. The picture shows the length on the epiglottis (a) and the distance of the perpendicular from the fixed point on the pharyngeal roof to the reference length on the epiglottis (b). Both distances were measured, and their quotient was calculated as described by Go et al. (PE ratio = *b*/a) (Barton et al., 2023; Go et al., 2014).

Freeze frames were used, and structures on the dorsal pharynx (such as follicles or vascular patterns) and the epiglottis were determined. These anatomical structures were used as fixed points. The distance of the perpendicular from the fixed point on the pharyngeal roof to the reference length on the epiglottis (b) was measured, and their quotient was calculated. This quotient has been called pharynx‐epiglottis (PE) ratio (PE = *b*/*a*; Figure 3) (Barton et al., 2023, Go et al., 2014).

As stated in previous studies, the width of the epiglottis (a) remains the same during exercise and head–neck positions in healthy horses and can therefore be used as reference length for the epiglottis (Barton et al., 2023, Go et al., 2014).

### 2.6 Statistical analysis

Due to the presence of repeated measures (every individual horse was tested with two different nosebands, loose and tight), generalized linear mixed effects models with noseband type as a single predictor and the individual animal as a random effect were chosen for analysis. The following parameters became response variables in the separate univariable models: swallowing counts, secretion grade, full adduction of the arytenoid cartilages counts, deviation of the aryepiglottic folds counts, soft palate instability counts, pharyngeal collapse grade, pharyngeal epiglottic ratios, dorsal displacements of the soft palate counts, and ridden horse pain ethogram scores.

The following model assumptions were checked: (1) the normality of residuals was checked by the Shapiro–Wilk normality test, (2) the homogeneity of variances between groups was checked with Bartlett test, and (3) the heteroscedasticity (constancy of error variance) was checked with the Breusch–Pagan test. In case assumptions were satisfied, generalized linear mixed effects models were used (R pac–age—lmer). In case assumptions were violated, robust linear mixed effects models were applied (R pac–age—robustlmm). Additionally, both linear and robust linear models were compared amongst each other using six main performance quality indicators: Akaike information criterion (AIC), Bayesian information criterion (BIC), conditional coefficient of determination *R* ^*2*^, marginal coefficient of determination *R* ^*2*^, the intraclass correlation coefficient (ICC), and root mean square error (RMSE). The model showing the best combination of predictive (AIC and BIC) and fitting power (explanatory, *R* ^*2*^, ICC, and RMSE) was preferred. The contrasts (differences) between tight and loose nosebands were assessed after model‐fitting by the estimated marginal means (R pac–age—emmeans). Results with a *p*‐value < 0.05 were considered statistically significant. No correction for multiple comparisons was conducted in order to reduce the probability of type II errors due to a small sample size. Data analysis was performed using R 4.3.1 statistical software (2023‐06‐16, R Foundation for Statistical Computing).

## 3 RESULTS

### 3.1 Video analysis of overground videos, PE ratio

The horses tolerated the procedure well, regardless of whether they were ridden with tight or loose nosebands first (randomly assigned). Results of the evaluated parameters are shown in Tables 2 and 3.

**TABLE 2:** Results of descriptive statistics of the used parameters in horses ridden with loose and tight nosebands (*n* = 16).

Parameter	Characteristic/unit	Loose (n = 16), mean (±SD)	Tight (n = 16), mean (±SD)
Swallowing	Counts	4.56 (3.14)	5.77 (4.38)
Secretion	Grade (0‐4)	1.50 (0.89)	3.13 (0.96)
Pharyngeal collapse (Boyle et al., 2006)	Grade (1‐4)	0.69 (0.87)	1.88 (1.54)
Full adduction of arytenoid cartilages	Counts	11 (12)	10 (7)
Axial deviation of aryepiglottic folds (ADAF)	Counts	0.29 (0.73)	1.33 (1.44)
Soft palate instability	Counts	278 (191)	330 (248)
Dorsal displacement of the soft palate (DDSP)	Counts	0	0.94 (2.08)
PE ratio (Go et al., 2014)	Ratio	1.28 (0.16)	1.30 (0.17)
RHpE Score (Dyson et al., 2018)	Score	4.75 (2.21)	6.40 (2.20)

*Note*: Parameters were either counted or graded during a defined period of the standardized exercise protocol (10 min, trot—canter). For specific definitions, see Table 1.

Abbreviations: PE, pharynx‐epiglottis; RHpE, ridden horse pain ethogram.

**TABLE 3:** Results of the univariable mixed effect models. *p*‐Value was considered significant when < 0.05, confidence level used: 0.95.

Parameter (unit)	OR	95% CI	p value
**Noseband tight ‐ loose**			
Swallowing (counts)	0.94	0.47, 2.35	0.191
Secretion (grade 0‐4)	1.62	0.98, 2.27	<0.001
Pharyngeal collapse (grade 1‐4) (Boyle et al., 2006)	1.19	0.43, 1.95	0.005
Full adduction of arytenoid cartilages (counts) (Lane et al., 2006)	0.27	5.97, 6.51	0.933
Axial deviation aryepiglottic folds (counts) (Lane et al., 2006)	0.98	0.28, 1.69	0.011
Soft palate instability (counts) (Lane et al., 2006)	51.7	129, 232	0.548
PE ratio (ratio) (Go et al., 2014)	0.02	0.10, 0.14	0.750
RHpE score (score) (Dyson et al., 2018)	1.55	0.84, 2.27	<0.001

*Note*: Parameters were either counted or graded during a defined period of the standardized exercise protocol (10 min, trot—canter). For specific definitions, see Table 1.

Abbreviations: CI, confidence interval; OR, odds ratio; PE, pharynx‐epiglottis; RHpE, ridden horse pain ethogram.

**FIGURE 4:**
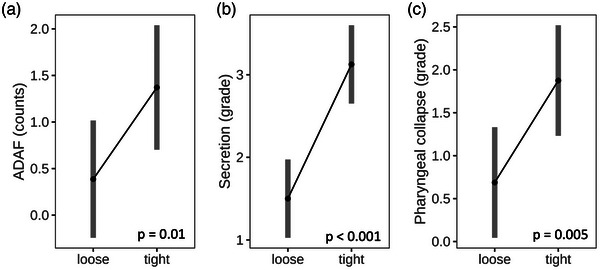
The parameters showing significant differences between horses ridden with loose versus tight nosebands: axial deviation of aryepiglottic folds (ADAF) counts (mean ± SD loose 0.29 ±0.73 vs. tight 1.33 ± 1.44), grades of secretion (mean ± SD loose 1.5 ± 0.89 vs. tight 3.13 ± 0.96) and pharyngeal collapse (mean ± SD loose 0.69 ± 0.87 vs. tight 1.88 ± 1.54).

While the PE ratio did not change significantly in horses ridden with loose versus tight nosebands, there were significant increases in grade of secretion (mean ± SD loose 1.5 ± 0.89 vs. tight 3.13 ± 0.96; *p* < 0.001), grade of pharyngeal collapse (mean ± SD loose 0.69 ± 0.87 vs. tight 1.88 ± 1.54; *p* = 0.005), and ADAF counts (mean ± SD loose 0.29 ±0.73 vs. tight 1.33 ± 1.44; *p* = 0.01) (Figure 4) in horses ridden with tight nosebands. There was no statistically significant difference between the application of a tight noseband versus a loose noseband on measures of swallowing counts (*p* = 0.19), full adduction of the arytenoid cartilages counts (*p* = 0.93), and soft palate instability counts (*p* = 0.55). Three horses repeatedly showed DDSP when ridden with tight nosebands, whereas no horse ridden with loose noseband had DDSP (Tables 2 and 3)

### 3.2 Video analysis of ridden horses and application of the RHpE

**FIGURE 5:**
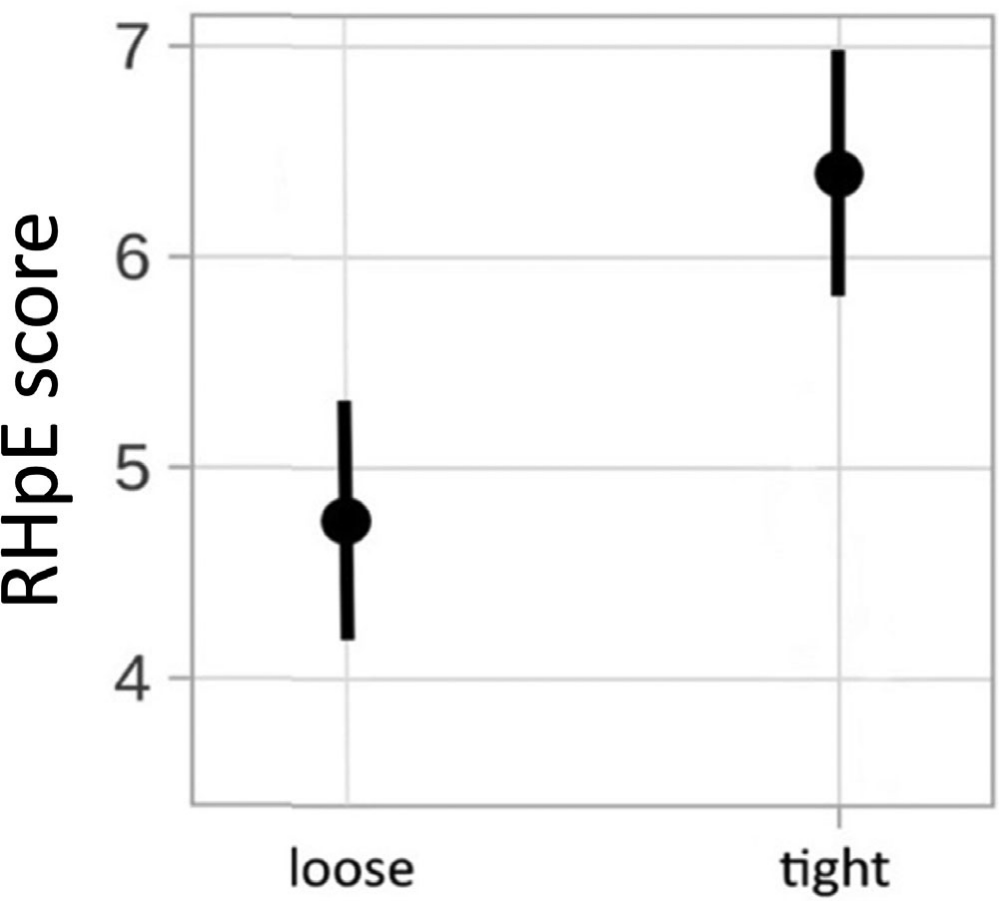
Ridden horse pain ethogram (RHpE) scores in horses ridden with loose versus tight nosebands. RHpE scores were significantly higher in horses ridden with tight nosebands (tight nosebands mean 6.40 ± SD 2.20 vs. loose nosebands 4.75 ± SD 2.21; *p* < 0.001).

There was no RHpE score above 8 indicating musculoskeletal pain; however, the RHpE scores were significantly higher in horses ridden with tight nosebands (mean score in horses ridden with tight nosebands 6.40 (±SD 2.20) versus mean score in horses ridden with loose nosebands 4.75 (±SD 2.21; *p* < 0.001; Figure 5). While the group with loose nosebands mainly demonstrated behavioural changes such as head tilting, carrying the head in front of the vertical, and opening the mouth, the horses ridden with tight nosebands mainly showed behavioural patterns such as carrying the head behind the vertical, ears turned back, eyelids closed, a visible sclera, and intense staring (detailed results can be found in Scholler et al. 2023). Head carriage behind the vertical (RHpE parameter No. 4), which also could influence pharyngeal structures, was displayed by 56% of the horses (9/16) with tight nosebands during this study compared to 44% of the horses (7/16) ridden with loose nosebands.

## 4 DISCUSSION

The aim of the study was to use overground endoscopy to evaluate changes in upper airway function when a tight noseband is fitted. When parameters regarding the pharyngeal region were evaluated, there was significantly more visible secretion in horses ridden with tight nosebands. In a normal horse, the accumulation of secretions in front of the larynx would probably prompt a swallowing response, which might have been inhibited by the tight noseband. In ridden horses, the tight noseband restricts tongue movements, which are needed for swallowing and also dissipate pressure from the bit, by pressing the bit against the tongue (Doherty, Casey, et al., 2017, Fenner et al., 2016, Mcgreevy et al., 2012). Swallowing was neither increased nor decreased in horses with tight nosebands during riding, but the post‐inhibitory rebound response of this parameter was not evaluated in this study. Other studies have shown a decrease in swallowing activity as noseband tightness increased, as well as a significant post‐inhibitory rebound response (Duke, 2017).

Pharyngeal collapse was seen in horses ridden with loose and tight nosebands, and there was a significant increase in the tight noseband group. Studies showed that pharyngeal collapse is likely associated with head and neck position and rider intervention (Cehak et al., 2010). As a head position behind the vertical was more frequently seen in horses ridden with tight nosebands, this might be an explanation of the situation. The pressure of the tongue basis against the soft palate and the epiglottis in horses with tight nosebands that cannot swallow despite increased secretion could also be a factor leading to pharyngeal collapse. Another study found that dressage horses ridden in flexed position are more often affected by dorsal displacements of the soft palate (Franklin et al., 2006). These displacements of pharyngeal structures (e.g., DDSP) lead to a decrease in airflow and an increase in expiratory resistance in the upper airways (Rehder et al., 1995). The reason for increased ADAF counts in the tight noseband group remains unclear. The condition only arises during exercise and can occur isolated or in combination with other forms of dynamic airway collapse (Franklin, 2008). In the case of combined forms, loss of arytenoid abduction or elevation of the epiglottis may cause reduced tension in the aryepiglottic folds, but the cause for isolated forms is unknown (Martin et al., 2000, Tan et al., 2005). As there are no muscular elements stabilizing these folds, flaccid tissue may be influenced by the suction effect during inspiration. A more negative inspiratory pressure may additionally lead to collapse of pharyngeal structures but seems to be an unlikely cause of isolated ADAF given the unremarkable findings in pharyngeal diameter (Lane et al., 2006).

The RHpE represents a system to assess musculoskeletal pain, but many of the used parameters are also associated with increased pain and stress levels of ridden horses (Ladewig et al., 2022, Uldahl & Clayton, 2019). By using the RHpE, Dyson et al. found that apart from musculoskeletal pain, stress and other suboptimal welfare conditions can result in the same behaviours as listed in the RHpE (Dyson & Pollard, 2020, Dyson et al., 2018, 2022; Dyson & Pollard, 2021a, 2021b; Dyson & Ellis, 2022). The RHpE comprises 24 aspects and behaviours (Dyson et al., 2018, Weller et al., 2020), which are recorded and evaluated over a period of about 10 min (Dyson et al., 2018, Weller et al., 2020) in ridden horses, as performed in this study. The RHpE for assessing pain‐associated behaviour has already been used at international Grands Prix in dressage and other major horse events (Dyson & Pollard, 2021b, Dyson & Pollard, 2022). An RHpE score of more than 8 out of 24 was considered representative of the presence of lameness or painful musculoskeletal disorders (Dyson et al., 2018). In addition to musculoskeletal diseases, stress and other factors that can affect the well‐being of the horse can also trigger similar behaviours as listed in the RHpE, but a cut‐off score has not been elucidated yet. The horses ridden with tight nosebands in this study predominantly showed behavioural patterns associated with the so‐called ‘learned helplessness’ (Hall et al., 2008, Hall et al., 2013), which is interpreted as a reaction to a stressor that the horse can only face with resignation.

Tight and restrictive nosebands are still common in equestrian sports despite the equestrian associations’ strive for regulations during competitions (FEI Inside, 2023). It has been shown that there is a widespread tendency to tighten the noseband to a substantially higher level of tightness than that suggested in equestrian guidelines (Clayton & Williams, 2022, Doherty et al., 2016, Doherty, Conway, et al., 2017, Merkies et al., 2022, Uldahl & Clayton, 2019, Visser et al., 2019, Weller et al., 2020). The use of these tight nosebands is controversial, and various studies aimed to investigate their various effects on the horse more carefully. Chewing movements indicating suppleness that are desired in equitation are almost impossible with tight nosebands (Clayton & Williams, 2022, Doherty et al., 2016, 2017, Merkies et al., 2022, Visser et al., 2019). In addition, direct physical damage can also occur because of the exposure to a noseband that is too tight. This implies swelling around the bridge of the nose, hair loss, or loss of colour of the hair under the noseband or sores (Casey et al., 2013, Perruccio & Scofield, 2017). Nosebands may compromise vascular perfusion and may cause nerve and bone damage according to earlier studies (Crago et al., 2019, Doherty, Casey, et al., 2017, Duke, 2017, Pérez‐Manrique et al., 2020, Uldahl & Clayton, 2019, Weller et al., 2020). Inside the oral cavity, tight nosebands may lead to soft tissue damage as the cheeks are pressed against the teeth (Kienapfel & Preuschoft, 2010, Perruccio & Scofield, 2017, Visser et al., 2019).

Many studies have also emphasized on the effects tight nosebands have on the horses’ stress response and its implications on equine welfare. Systemic stress responses are indicated by increases in eye temperature and heart rate and decreases in heart rate variability (McGreevy, 2015, Rietmann et al., 2004, Valera et al., 2012), while behavioural responses such as chewing, licking, and yawning were shown more frequently by the horses following the removal of the noseband as a sign of post‐inhibitory rebound response (Freire et al., 2009, Nicol, 1987). In horses, a post‐inhibitory rebound response represents a negative welfare state as they build up motivation and are deprived of the desired behaviour (Ashley et al., 2005, Freire et al., 2009, Mclean & Mcgreevy, 2010, Nicol, 1987). The effect on welfare is more marked when behaviours with post‐inhibitory rebound responses are prevented than when behaviours without these rebound responses are suppressed (Freire et al., 2009, Rietmann et al., 2004).

While the association between tight nosebands and stress in horses has been the subject of various studies (Doherty, Casey, et al., 2017, Fenner et al., 2016), the stress effect elicited by the overground endoscope has not been further investigated in this context. Various studies found that horses showed very good acceptance of endoscopic examination during exercise (Pollock & Reardon, 2009, Tamzali et al., 2008, Zebisch et al., 2014). Nevertheless, riding with the overground endoscope is believed to produce at least some form of stress and discomfort in horses due to foreign body irritation in the nose and throat. In a study by Scholler et al. (2023), the horses ridden with overground endoscope showed higher cortisol levels than the ones ridden without, regardless of the tightness of the noseband (3). As both groups (loose and tight nosebands) in this study were ridden with the same type of endoscope, and the order of setting (tight or loose) was randomized for the individual horse, the stress response with regard to the endoscope should not have affected results.

The width of the epiglottis is assumed to remain constant in healthy horses during exercise based on preceding studies (Barton et al., 2023, Cehak et al., 2010, Go et al., 2014, Van Erck, 2011). Therefore, measuring the PE ratio proved to be more reliable than the dorsoventral pharyngeal diameter, which is substantially affected by head–neck position in horses at rest (Cehak et al., 2010, Go et al., 2014). The influence of head–neck position on PE ratio has been investigated in many studies, whereas there are no studies on the impact of tight nosebands on the PE (Hanche‐Olsen et al., 2010, Petsche et al., 1995, Strand et al., 2009, Van Erck, 2011). As stated in other studies, this study confirmed that the PE ratio remained constant regardless of the noseband setting the horses were ridden with.

ADAF, which significantly decreases the upper airway diameter in affected horses, was seen more frequently in horses ridden with tight nosebands. A study by Tilley et al. (2023) stated that a 15° variation in poll flexion (just behind the vertical) resulted in an increased occurrence of ADAF, palatal instability or dysfunction, and collapse of various pharyngeal structures. As the tight noseband group in this study showed an increased occurrence of ADAF, this may partly be due to the head carriage behind the vertical. The changes in head carriage between the different noseband settings can be interpreted as a sign of stress and discomfort (Dyson & Ellis, 2022, Dyson et al., 2018). As there is no basic support through cartilage or bone in the caudal part of the nasopharynx in horses, its stability is dependent on the muscular tone. Based on the findings in this study, the tight noseband leads to collapse of pharyngeal structures possibly due to compromised oral behaviour in the horses. The ventral reduction in the pharyngeal diameter originates from the retracted tongue being pressed against the soft palate from the oral cavity as tongue movements are restricted by the noseband. Studies have shown that horses use their tongues to control the distribution of bit pressure within the oral cavity (Engelke & Gasse, 2003, Manfredi et al., 2009). As tight nosebands put pressure on the tongue by pressing the bit against it, the horse may try to escape this pressure by bulging up the tongue. Fighting against restrictive measures in the oral cavity may negatively influence pharyngeal diameter and lead to disturbances in the upper airways (Ahern, 2019, Doherty, 2016). In a study on the influence of tongue ties on the upper airways, horses showed DDSP four times more often with a tongue tie applied than without it (Barton et al., 2023). This is in concordance with the findings in this study as some of the horses showed DDSPs with tight nosebands, whereas none of the horses had a DDSP when ridden with a loose noseband. However, another study found that tight nosebands restrict palatal instability and movements of the soft palate (Ahern, 2018).

## 5 CONCLUSION

In this study, very tightly fastened nosebands led to increased stress in the horses based on the RHpE parameters and also resulted in more visible secretion and collapse of pharyngeal structures. As tight nosebands have a significant impact on horses’ upper airways, further regulations or enforcement of the regulations that are already in place are recommended regarding noseband use.

## AUTHOR CONTRIBUTIONS

**Dominik Scholler**: Conceptualization; Data curation; Formal analysis; Investigation; Validation; Visualization; Writing—original draft preparation. **Jana Wittenberg**: Data curation; Investigation. **Yury Zablotski**: Formal analysis; Software; Validation; Visualization; Writing—review and editing. **Anna May**: Conceptualization; Data curation; Investigation; Methodology; Writing—original draft preparation; Project administration; Resources; Supervision; Writing—review and editing.

## CONFLICT OF INTEREST STATEMENT

The authors declare no conflicts of interest.

## FUNDING INFORMATION

This study did not receive funding.

### ETHICS STATEMENT

The authors confirm that the ethical policies of the journal, as noted on the journal's author guidelines page, have been adhered to, and the appropriate ethical review committee approval has been received. The study protocol was approved by the animal ethics committee of the government of Bavaria (Oberbayern), Munich, Germany (approval AZ ROB‐55.2‐2532.Vet_02‐21‐100, February 2022).

### PEER REVIEW

The peer review history for this article is available at https://www.webofscience.com/api/gateway/wos/peer-review/10.1002/vms3.1478.

## Supporting information



Supporting Information

## Data Availability

The data that support the findings of this study are available from the corresponding author upon reasonable request.
