# Supplementary material for: Do tight nosebands have an effect on the upper airways of horses?
Source: Vet Med Sci. 2024 Jun 17;10(4):e1478. doi: 10.1002/vms3.1478 (PMC11182419; doi:10.1002/vms3.1478)
Supplement: Supplementary file 1 — Supporting Information [file VMS3-10-e1478-s001.docx]

**10 min standardized exercise test for noseband study (tight versus loose)**

| 1. |  | 10 minutes warm up, then working trot |
| --- | --- | --- |
| 2. | C  E-X | Track left  Half circle left 10m, returning to track at H |
| 3. | B-X | Half circle right 10m, returning to track at M |
| 4. | C  C | Circle left 20 m in rising trot  Working trot |
| 5. | H-P  P | Change rein, lengthen strides in trot  Working trot |
| 6. | A | Medium trot |
| 7. | V-R  R | Change rein, working trot  Medium trot |
| 8. | M  C | Working trot  Working canter left lead |
| 9. | E | Circle left 15m |
| 10. | F-X-H  X | Change rein  Working trot |
| 11. | C | Working canter right lead |
| 12. | B | Circle right 15m |
| 13. | A | Working trot |
| 14. | K-R  R | Change rein, lengthen strides in trot  Working trot |

Standardized exercise protocol inspired by 2022 USEF First Level Test 1 or A level test (FN, Germany). Duration: 5 minutes, was ridden twice by the riders to have 10 minutes of standardized exercise test at first level.
